# 2700. Reducing the Dose of Mycophenolate in Solid Organ Transplant Recipients Diagnosed with SARS-CoV-2 Infection Increases the Risk of Severe COVID-19

**DOI:** 10.1093/ofid/ofad500.2311

**Published:** 2023-11-27

**Authors:** Marion Gillcrist, Stephen Waller, Ryan M Taylor, Nathan C Bahr, Diane M Cibrik, Zubair Shah, Albert Eid

**Affiliations:** University of Kansas Medical Center, Wichita, Kansas; University of Kansas, Kansas City, Kansas; University of Kansas School of Medicine, Kansas City, Kansas; University of Kansas Medical Center, Wichita, Kansas; University of Kansas Medical Center, Wichita, Kansas; University of Kansas Medical Center, Wichita, Kansas; University of Kansas Medical Center, Wichita, Kansas

## Abstract

**Background:**

Most of the available COVID-19 outcome data in solid organ transplant (SOT) recipients does not suggest a higher mortality rate in the SOT population. Though theoretically immunosuppressive medications might be associated with worse outcome, its immunomodulatory effect might be beneficial.

**Methods:**

We retrospectively analyzed the effect of dose-reduction of mycophenolate, tacrolimus, and prednisone on the severity of COVID-19 in SOT recipients diagnosed with COVID-19 at the University of Kansas Medical Center between January 1, 2020 and December 31, 2022. Severe COVID-19 was defined by development of hypoxia while the patient was on room air during a 21-day period following COVID-19 diagnosis. To evaluate the effect of dose changes after the diagnosis of COVID-19 on the odds of developing severe COVID-19, we subset our data based on whether patients were taking mycophenolate (n = 449), tacrolimus (n = 537) or prednisone (n = 386) at the time of diagnosis. Within each subgroup, we fit a logistic regression model with severe COVID-19 as the response variable and adjusted for the relevant immunosuppressant and a set of covariates (age, sex, race, body mass index, hypertension, diabetes mellitus, prespecified time period of COVID-19 diagnosis during the pandemic, chronic heart disease, chronic lung disease, malignancy, end stage renal disease, cirrhosis, smoking, receipt of COVID-19 vaccine, and receipt of SARS-CoV-2 monoclonal antibodies).

**Results:**

In our SOT population, 570 patients were diagnosed with COVID-19 (458 kidney, 73 liver, 54 heart, 43 pancreas, and 8 lung recipients). The median patient age was 54 years, 59% were male, and 70% were white. After adjusting for other covariates, a mycophenolate dose-reduction of < 50% was associated with an odds ratio of 2.01 (95% CI: 1.03 - 3.90; p = 0.04) for developing severe COVID-19. Similarly, decreasing the dose of mycophenolate by ≥ 50% was associated with an odds ratio of 2.65 (95% CI: 1.09 - 6.41, p = 0.03) for developing severe disease. Tacrolimus dose reduction did not increase the risk of severe COVID-19, nor did a dose increase or decrease of prednisone.

**Conclusion:**

A mycophenolate dose reduction when a SOT patient is diagnosed with COVID-19 should be discouraged, especially if the patient has mild disease.

**Disclosures:**

**All Authors**: No reported disclosures

